# On the relationship between spectroscopic constants of diatomic molecules: a machine learning approach

**DOI:** 10.1039/d1ra02061g

**Published:** 2021-04-19

**Authors:** Xiangyue Liu, Gerard Meijer, Jesús Pérez-Ríos

**Affiliations:** Fritz-Haber-Institut der Max-Planck-Gesellschaft Faradayweg 4-6 14195 Berlin Germany jperezri@fhi-berlin.mpg.de

## Abstract

Through a machine learning approach, we show that the equilibrium distance, harmonic vibrational frequency and binding energy of diatomic molecules are related, independently of the nature of the bond of a molecule; they depend solely on the group and period of the constituent atoms. As a result, we show that by employing the group and period of the atoms that form a molecule, the spectroscopic constants are predicted with an accuracy of <5%, whereas for the A-excited electronic state it is needed to include other atomic properties leading to an accuracy of <11%.

## Abstract 2

Through a machine learning approach, we show that the equilibrium distance, harmonic vibrational frequency and the binding energy of diatomic molecules are universally related, independently of the nature of the bond of a molecule.

## Graphical abstract

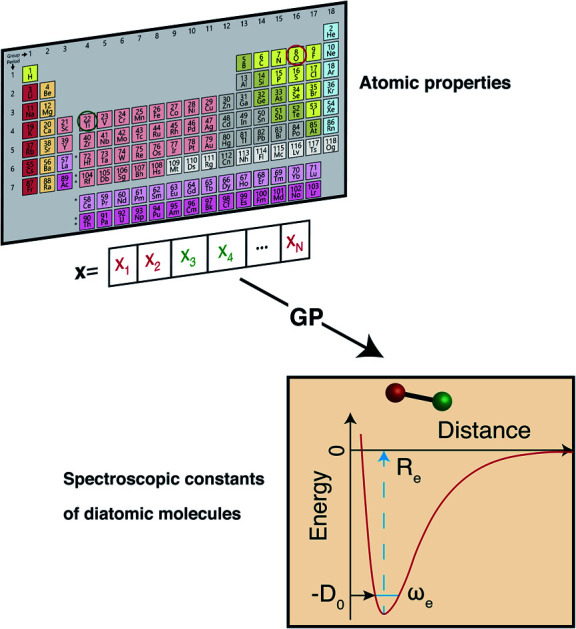

## 1 Introduction

Early in the history of molecular spectroscopy, when it became a discipline within chemical physics in the 1920's,^1^ some intriguing empirical relationships between different spectroscopic properties were observed.^2–4^ In particular, it was found that the equilibrium distance, *R*_e_, and the harmonic vibrational frequency, *ω*_e_, were correlated in diatomic molecules. As the field evolved, the relationship between *R*_e_ and *ω*_e_ became more evident, and more empirical relations between spectroscopic constants were identified.^5–12^ However, these empirical relationships were typically only valid for specific atomic numbers or groups of the constituent atoms. These results motivated the development of realistic diatomic molecular potentials^4,13–17^ and triggered the physical chemistry community to think about the “periodicity” of diatomic molecules.^18^

The development of quantum chemistry helped to shed some light on the physics behind empirical relationships between spectroscopic constants. In particular, thanks to the application of the Hellmann–Feynman theorem, it was possible to connect *ω*_e_ directly with the electronic density at *R*_e_.^19–22^ As a result, a first principles-based explanation (containing a few free parameters), of the observed empirical relations between spectroscopic constants appeared.^23–30^ Nevertheless, the obtained relations based on the electronic density were only valid for subsets of molecules. To date, it has not been possible to find general relations for spectroscopic constants of diatomic molecules in terms of the properties of their constituent atoms.

The accuracy of quantum chemistry methods relies on (finite) basis sets optimized for each element under certain bounds. At the same time, an accurate description of the system's electronic structure is required, which is achieved through a hierarchy of different treatments of the electron correlation.^31,32^ On the other hand, the widely-used (Kohn–Sham) density functional theory (DFT) methods require accurate electron exchange–correlation density functionals. The non-empirical density functionals are derived under certain constraints, some with several free parameters,^33–36^ while the semi-empirical density functionals employ more flexible functional forms with (sometimes even several tens of) coefficients fitted to various experimental or theoretical reference properties.^33,37^ Machine learning (ML) methods, on the other hand, discover the underlying relationships from data (the so-called “training set”) and build up models on top of them. These models can be quantitatively predictive for other systems that follow similar underlying physics. More importantly, they provide possibilities for discovering relationships between the different properties of the system under consideration.^38,39^

This work shows that the relationship between spectroscopic constants of heteronuclear diatomic molecules is general for most kinds of molecules at hand. Our findings rely upon applying state-of-the-art ML models to an orthodox dataset of experimental spectroscopic constants for diatomic molecules. In particular, we apply the Gaussian process (GP) regression model^40^ to predict *R*_e_, *ω*_e_, and the binding energy, *D*_0_, as a function of the group and period of the constituent atoms. Similarly, our model can predict *R*_e_ and *ω*_e_ for the A-excited electronic state of a given molecule. Our findings generalize the idea that some of the system's chemical properties depend on the atoms' group and period. Indeed, the periodicity of elements has long been used to predict chemical compounds' properties intuitively at a qualitative level. However, the correlations between the chemical properties and the constituent atoms' periodicity are not always straightforward, and such predictions can hardly be quantitative in most cases. On the contrary, our main result is quantitatively meaningful: it is possible to predict those spectroscopic constants with an accuracy of <5% for ground electronic states and <11% for the A-excited electronic state. More interestingly, by analyzing our models' outliers, we show that molecules showing a non-chemical bond nature like bi-alkali molecules and molecules containing first-row elements, such as HF, are more difficult to predict. However, the spectroscopic constants of molecules containing transition metals challenging for quantum chemistry methods can be adequately described.

## 2 The quest of relationships between spectroscopic constants of diatomic molecules

**Fig. 1:**
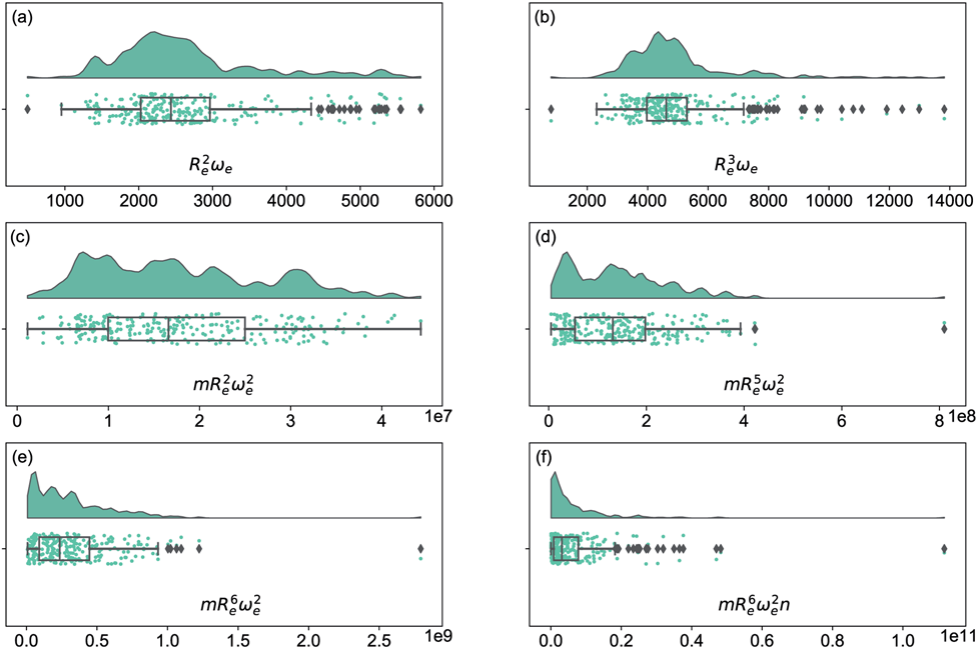
Distribution and box plots of *R*_e_^*a*^*ω*_e_^*b*^ with different powers combined with the reduced mass *m* and number of valence electrons *n*.

As soon as molecular spectroscopy became an essential tool to analyze molecules' unique fingerprints and more spectra of molecules were taken, approximate relationships were found between spectroscopic constants. As a result, it was postulated that the molecules' spectroscopic constants might be correlated based on empirical grounds. In particular, it was observed that the equilibrium distance and the harmonic vibrational frequency are related as *R*_e_^2^*ω*_e_^2^*m* = const in hydrogen halides,^2,41–43^ where *m* is the reduced mass of the molecule. This relationship was generalized as *R*_e_^*i*^*ω*_e_^2^*m* = const, the precursor of the well-known Badger's rule,^6^ where *i* is a natural number. On the other hand, after studying the spectra of 16 molecules, including homonuclear molecules and molecular ions, Mecke and Birge found that the expression *R*_e_^2^*ω*_e_ = const described the observed spectra better.^3,44^ In the same line, but using a given functional form for the interatomic interaction of a molecule, Morse proposed a relationship given as *R*_e_^3^*ω*_e_ = const.^4^ Finally, more involved relationships between the equilibrium distance and the vibrational harmonic frequency were proposed^17^ as *mR*_e_^6^*ω*_e_^2^*n*^*a*^, where *n* stands for the number of valence electrons, and *a* is a rational number. The results for a variety of the proposed empirical rules are shown in Fig. 1, where it is noticed that for a larger dataset, as the present one, none of the empirical relationships hold.

At the same time, more spectroscopic information of molecules became available, and more advanced and accurate quantum chemistry tools were developed. Therefore, it was possible to search for a first principle explanation of the empirically observed relationships between spectroscopic constants. In that endeavor, Parr and coworkers took the lead by looking at the electron density within a molecule as the source of the relationship between spectroscopic constants. The model assumes that the electron density mutually created by the one atom in the other atom is equal at the equilibrium distance, *i.e.*, at the sum of two atomic radii. In particular, the electron density of atom 1 at the position of atom 2, within a molecule, is given by^27^

1 *ρ*_1_(2) = *CZ*_1_ exp(−*ξR*_1_),

where *C* is a fitting parameter and *ξ* represents the decay constant of the electron density. Within this model, one finds a relationship between the atomic numbers of the two atoms, *Z*_1_ and *Z*_2_, and the equilibrium internuclear distance *R*_e_ of a diatomic molecule as^27,29,30,45^

2 *Z*_1_*Z*_2_ = *A* exp(*ξR*_e_),

where *A* is a free parameter. According to this relationship, *R*_e_ depends linearly on log(*Z*_1_*Z*_2_) as

3 *R*_e_ = *ξ*^−1^ log *Z*_1_*Z*_2_ − *ξ*^−1^ log *A*.

However, the performance of this relationship has only been checked for molecules with atoms coming from the same group of the periodic table.^29^

Anderson, Parr and coworkers also suggested a relationship between *ω*_e_ and *R*_e_^29^ as

4 *mω*_e_^2^ = 4π*CZ*_1_*Z*_2_*e*^−2*R*_e_^,

based on the Born–Oppenheimer approximation, the electron density of eqn (1) and the Hellmann–Feynman theorem. From eqn (4) it is possible to express the harmonic vibrational frequency in terms of the equilibrium distance and atomic properties as5
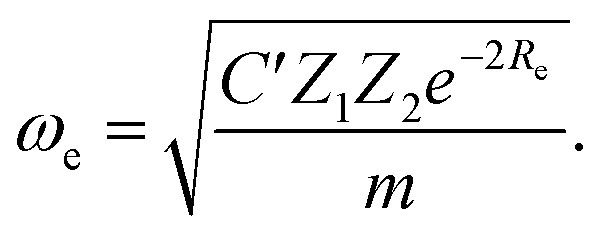


In the same vein, following the relationship between the equilibrium distance and the harmonic vibrational frequency, it is possible to find a relationship between the atomic number *Z*_*i*_, *R*_e_, and the dissociation energy *D*_e_, as^27,29,30,45^6
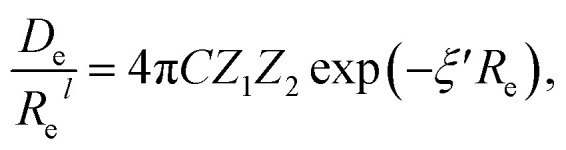
which can be rewritten as7
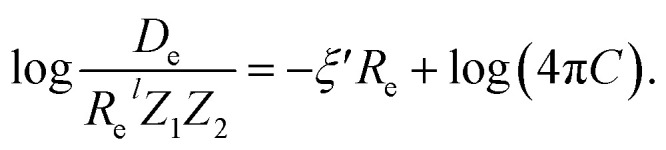


For the derivation of eqn (6) it must be assumed that *D*_e_ = *Amω*_e_^2^*R*_e_^*l*^ without any further justification.^30^ In eqn (7), *l* = 3 and *ξ*′ = 0.97. Eqn (7) has been tested in a dataset of 150 molecules leading to a good result, although no further characterization of the model performance was reported to objectively judge its quality. Finally, using the relation of the dissociation energy, *D*_e_, and the binding energy, *D*_0_,8
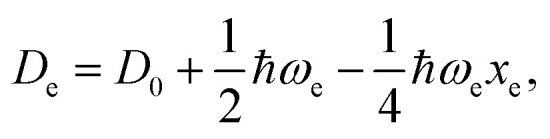
where *ω*_e_*x*_e_ represents the first anharmonic correction to the harmonic vibrational frequency, it should be possible to find a linear regression model for 
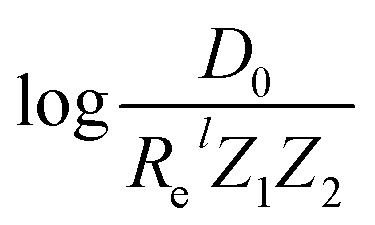
.

## 3 The dataset

In this work, we focus on heteronuclear molecules due to their relevance on laser cooling of molecules with applications in ultracold chemistry.^47–49^ The employed dataset contains the main spectroscopic constants: *R*_e_, *ω*_e_, and *D*_0_ for the ground electronic state of heteronuclear diatomic molecules. In particular, it contains the experimental values of *R*_e_, *ω*_e_ for 256 heteronuclear diatomic molecules taken from ref. 50–53, whereas the experimentally determined values of *D*_0_ are only available for 197 of them.

**Fig. 2:**
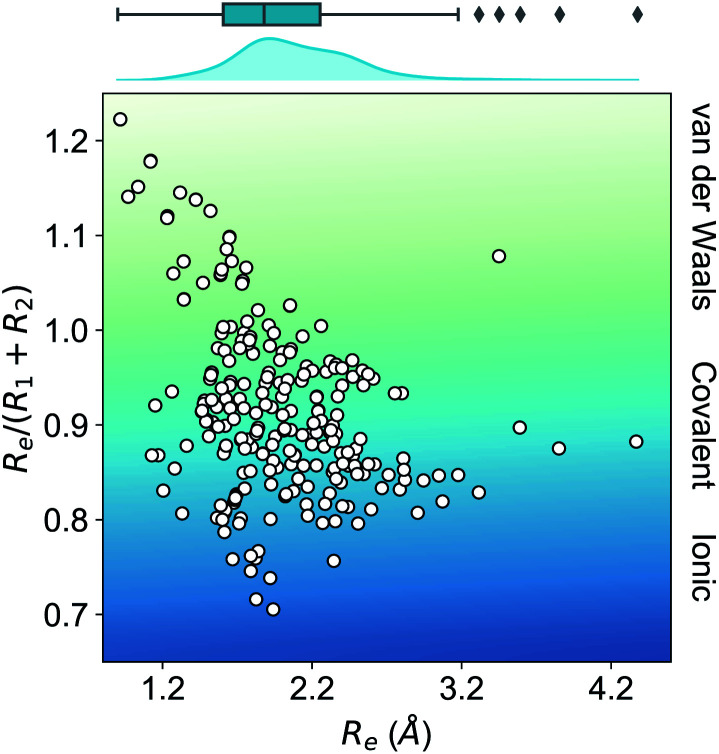
Ratio of the equilibrium distance, *R*_e_, to the sum of the atomic radii of the atoms forming a molecule, *R*_1_ + *R*_2_, *vs. R*_e_. The background color indicates the nature of the molecular bond in each of the molecules. The density in the upper part of the figure shows the kernel density distribution of *R*_e_. The box plot shows the minimum, the maximum, the sample median, and the first and third quarterlies of *R*_e_. The empirical atomic radii of the atoms are taken from ref. 46.

As far as we know, this is the most extensive dataset for experimental ground state properties of heteronuclear diatomic molecules. Fig. 2 shows the equilibrium distance's distribution and its ratio to the sum of the atomic radii of the constituent atoms, *R*_1_ + *R*_2_, for molecules within the dataset. Most molecules show an equilibrium distance between 1.4 Å and 3.8 Å, with a most probable value of 1.7 Å. Looking at the values of *R*_e_/(*R*_1_ + *R*_2_), it is clear that the molecules within the dataset have different bonds: covalent, van der Waals, and ionic.

**Fig. 3:**
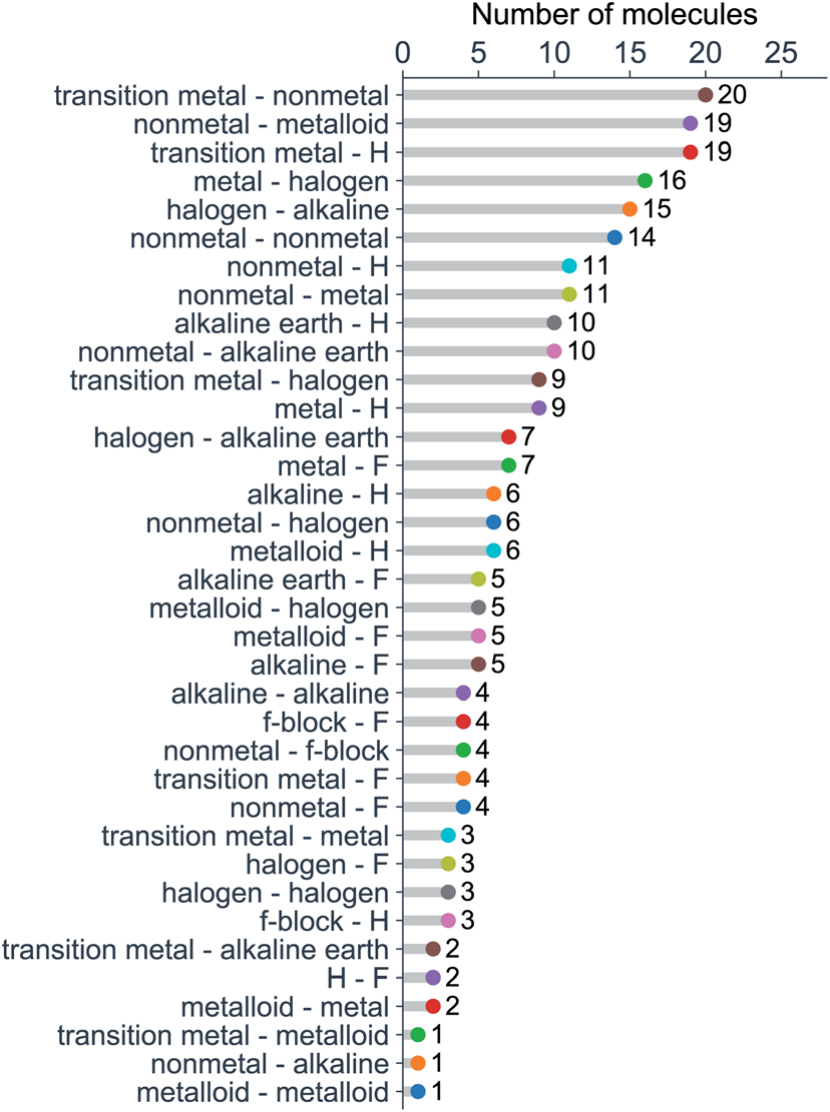
Molecules in the dataset classified by the types of their constituent atoms.

We have classified the dataset based on the types of constituent atoms within a molecule, and the results are shown in Fig. 3. As a result, we notice that the dataset mainly consists of various metal and non-metal halides, hydrides, and metalloid compounds. It is worth noticing that more than 20% of the dataset contains transition metal compounds, including f-block elements. Therefore, the present dataset is general since it goes beyond the main-group diatomic molecules and deals with some of the more intriguing and complex atoms from a chemistry standpoint.

In addition to the dataset mentioned above of the ground state properties, we also study 131 molecules whose *R*_e_, *ω*_e_ are available for the A-excited electronic state. The A-state dataset mainly consists of metal and non-metal compounds, including transition metal compounds and several f-block compounds.

## 4 Machine learning method

The quest for universal relationships between spectroscopic constants is related to the problem of how atomic and molecular properties describe a spectroscopic property of a molecule, *y* = *f*(**x**). Here, **x** = (*x*_1_, *x*_2_, …, *x*_*n*_), consists of different atomic properties of the constituent atoms or molecular properties, where *n* denotes the number of input features relevant for the problem at hand. Unlike traditional (non-)linear regression models, which assume a fixed form of function *f*(**x**), GP embraces a Bayesian perspective and presumes a prior distribution over the space of functions9
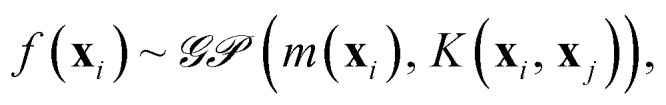
with a joint multivariate-Gaussian distribution, centered at *m*(**x**_*i*_) and characterized by the covariance function *K*(**x**_*i*_, **x**_*j*_), which specifies the correlation (or “similarity”) between data points.^40^

In this work, the spectroscopic properties *y* are modeled as10
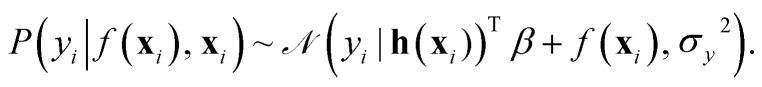
where the basis functions, **h**(**x**_*i*_), project {**x**_*i*_} to a new (higher dimensional) feature space with coefficients *β*, and *σ*_*y*_ includes the noise in the observations.^40,54^ The training set 
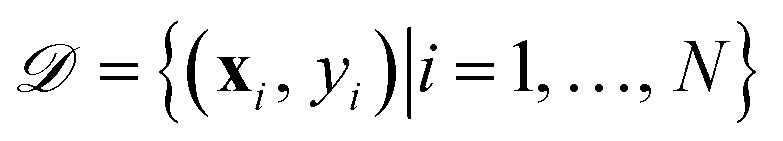
 with *N* observations, constrains the available distribution of functions through Bayes theorem, and the mean of the posterior distribution is used for prediction. The functional form of *K*(**x**_*i*_, **x**_*j*_) and **h**(**x**) can be selected according to the cross-validation performance of the models.

### 4.1 Model performance evaluation

In training and evaluating the regression models, as customary in ML, the ground state dataset is divided into training and test sets. The training set represents the set of molecules used for learning a given spectroscopic constant from the atomic properties of the constituents atoms. The test set is the set of molecules that have not been included in the learning procedure and hence are new to the regression algorithm. In learning the equilibrium internuclear distance, *R*_e_, and the harmonic vibrational frequency *ω*_e_, the training and test sets consist of 231 and 25 molecules, respectively. In learning 
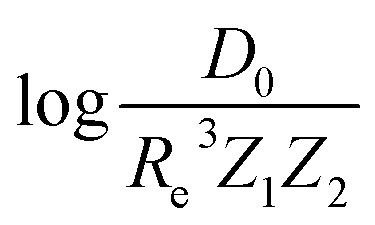
 the training/test splitting is 172/25. For learning *R*_e_ and *ω*_e_ for the A-excited electronic state, the training set consists of 106 molecules and the test set consists of 25 molecules.

The present dataset is relatively small from an ML perspective. When the dataset is split into training and test sets, the training set may not be representative. This may lead to a bias in the performance of the test set. To solve this problem, we have employed a Monte Carlo (MC) approach, in which the dataset is stratified into 25 strata based on the level of the true values of the labels (*R*_e_, *ω*_e_, and 
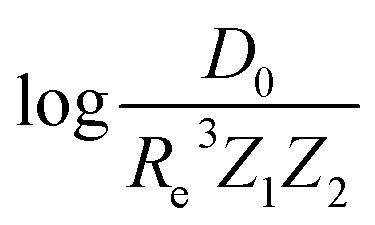
 in the present work).

**Fig. 4:**
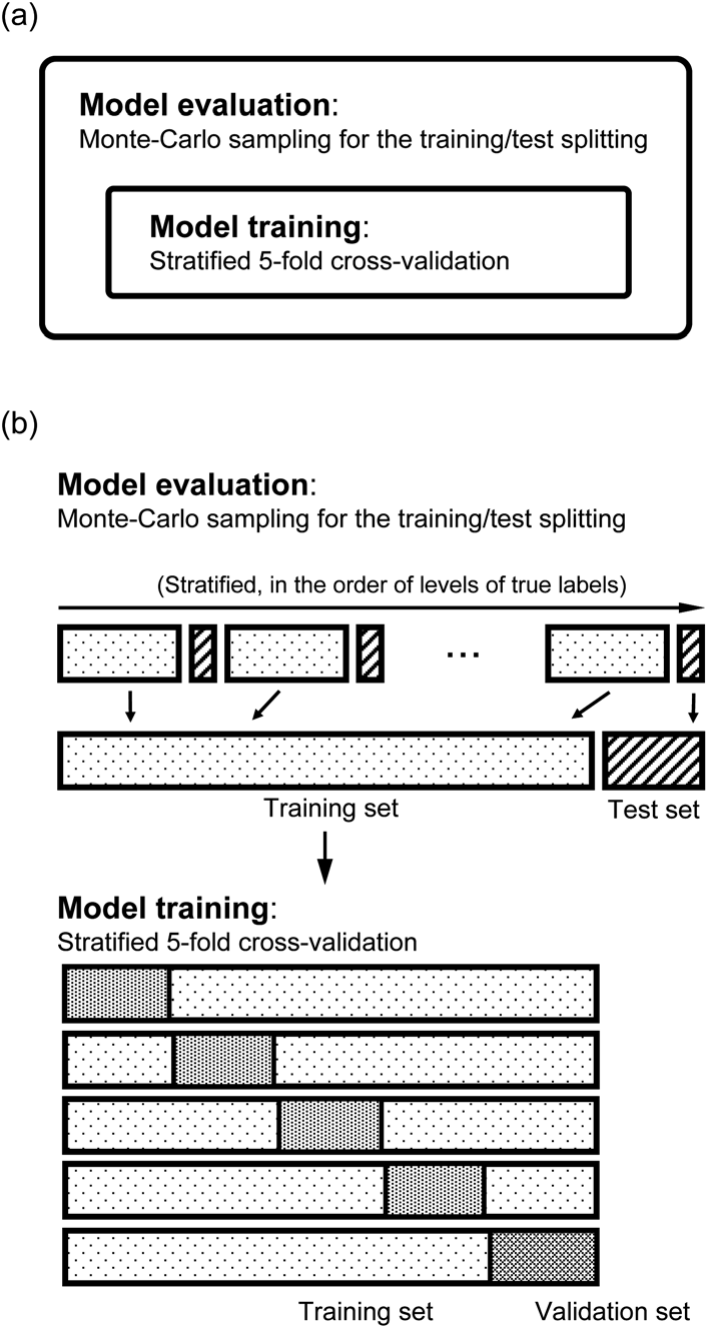
Scheme of the training/test set splitting in the model evaluation. (a) There are two loops: the outer loop for the model performance evaluation, and the inner loop for the training of model and hyperparameter optimization. (b) In the outer loop, the data are stratified based on the true values of the labels, and each stratum is randomly split into training and test sets. In learning the properties, the training sets are further split into training and validation sets to perform a stratified 5-fold cross-validation.

As shown in panel (a) of Fig. 4, we have two loops in the training and evaluation of the models. In the outer loop, we split the dataset into training set and test set. The training set is used to learn from the data and the test set is used for model evaluation. In the inner loop, we train the models with the training set, which is further split to perform a stratified 5-fold cross validation (CV) for the hyperparameter optimization. In particular, as shown in panel (b) of Fig. 4, in the outer loop, the training/test splittings are done by a Monte Carlo (MC) approach. Specifically, we randomly select 25 test molecules from the dataset, which is stratified into 25 strata based on the levels of the true values of the labels. The stratification helps to minimize the change of the proportions of the dataset compositions upon splitting.^55^ In each MC step, a regression model is trained and gives the predictions to the training set and the test set. Therefore, in this work we report the mean and standard deviation of the predictions for each molecule when they are used in the training and test sets from all the MC steps. In total, we evaluate our models with 1000 MC steps for the training/test splittings for the model performance evaluation, and 500 MC steps for generating the learning curves.

The performance of the models is evaluated by three different estimators. The first estimator is the mean absolute error (MAE) defined as11
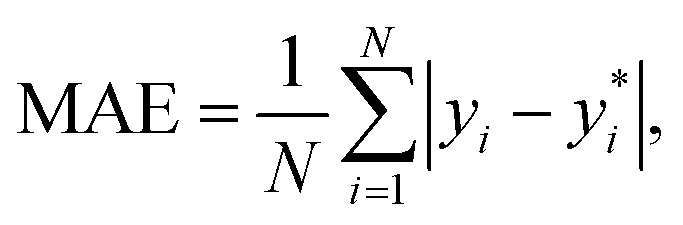
where 
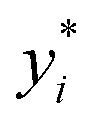
 are the true values, *y*_*i*_ are the predictions, and *N* is the number of observations. The second estimator is the root mean square error (RMSE), which is given by12
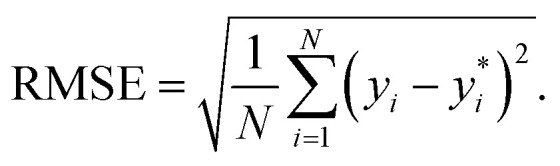


The last estimator is the normalized error *r*_E_, defined as the ratio of the RMSE to the range of *y*,13
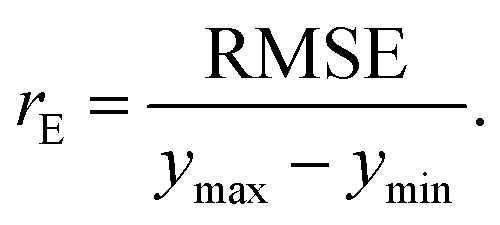


### 4.2 The learning curves

The learning curves show the training and test performance of a model as a function of the training set size *N*. From the learning curves it is possible to infer the performance of a model by looking at its bias and variance. Similarly, it is possible to understand if the model performance improves with the training set size. For each of the points in the learning curve, the training is performed with 500 different training/test splittings by the MC approach.

## 5 Results and discussion

### 5.1 Learning ground state spectroscopic constants

Fueled by the idea of periodicity of molecules (see, *e.g.*, ref. 18 and references in it), we use the group, *g*_*k*_, and period, *p*_*k*_, of the atoms within a molecule, *i.e.*, *k* = 1, 2, as input features for a GP regression model to predict different combinations of spectroscopic constants: *R*_e_, *ω*_e_ and 
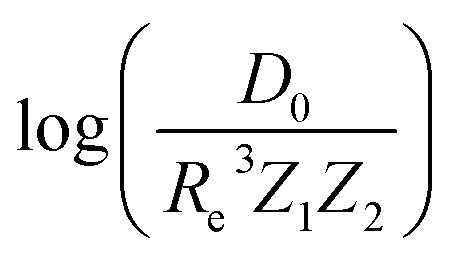
, as presented in Section 2. The training sets are permuted before feeding the learning algorithm to reproduce the permutational invariance of relevant properties upon exchanging two atoms in a molecule in the GP regression models.

**Fig. 5:**
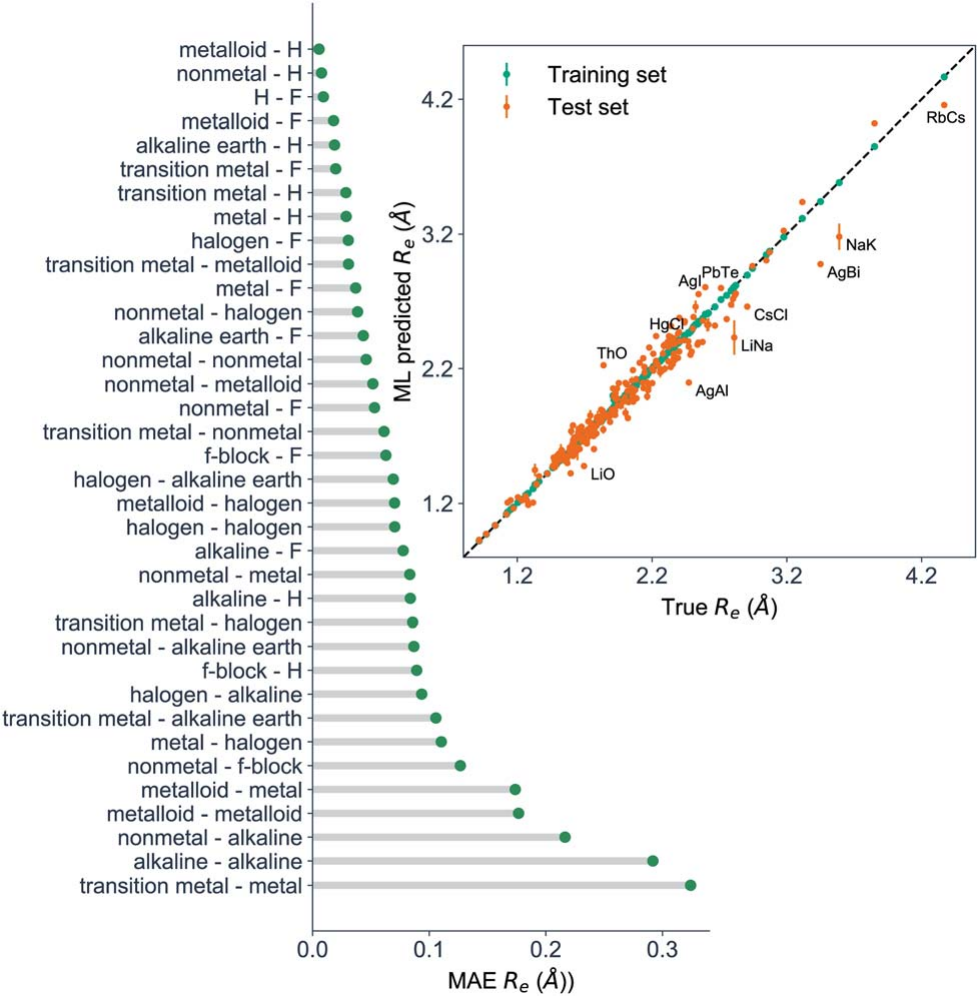
GP regression performance on predicting *R*_e_ using (*g*_1_, *g*_2_, *p*_1_, *p*_2_) as input features classified by the types of the constituent atoms. In particular, the MAE of the test set is reported. The inset shows the test set predictions of *R*_e_*versus* the true values. The values shown are the average of predictions from 1000 MC sampled training/test splittings. The GP regression model gives predictions of the test and training sets. Shown are the mean and standard derivation of each molecule's predictions when used as training data (green symbols) and test data (orange symbols).

The GP regression model performance of ground state *R*_e_ as a function of input features (*g*_1_, *g*_2_, *p*_1_, *p*_2_) is shown in Fig. 5, where the MAE associated with each of the distinct type of molecules is reported. As a result, most of the molecules are well described by our GP model, as confirmed in the inset of Fig. 5. In particular, it shows little dispersion of the predicted values concerning the true values except for a handful of molecules (transition metal–metal and bi-alkali molecules). To further quantify the GP regression model performance, we calculate the average RMSE of the predicted *R*_e_ on 1000 randomly selected test sets leading to 0.0968 ± 0.0070 Å (Table 1), and *r*_E_ = 2.80 ± 0.20%. Our results confirm that the model performance improves as the number of molecules in the training set, *N*, grows, as it is shown in the learning curve in panel (a) of Fig. 8. Indeed, it is not yet converged for *N* = 231, suggesting that the GP regression model can be further improved by learning more data in the training set.

**Table 1:** Regression model predictions of *R*_e_, *ω*_e_, and *D*_0_. *g*_*i*_ and *p*_*i*_ represent the group and period of the *i*-th atom, respectively. *g*^iso^_*i*_ stand for the group encoding the information of isotopes of hydrogen, and *p̄*, *ḡ* are the average of groups and periods of the two atoms, respectively

Property	Model	Feature	Test MAE	Test RMSE	Test *r*_E_ (%)
*R*_e_ (Å)	GPR	(*g*_1_, *g*_2_, *p*_1_, *p*_2_)	0.0662 ± 0.0037	0.0968 ± 0.0070	2.80 ± 0.20
	LR	log(*Z*_1_*Z*_2_)	0.2605 ± 0.0018	0.3591 ± 0.0006	10.41 ± 0.01
*ω*_e_ (cm^−1^)	GPR	(*R*_e_^−1^, *g*_1_, *g*_2_, *p*_1_, *p*_2_)	126.7 ± 2.1	207.2 ± 2.6	5.07 ± 0.06
		( 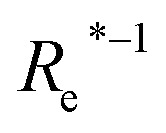 , *g*_1_, *g*_2_, *p*_1_, *p*_2_)^a^	152.5 ± 3.6	227.5 ± 4.6	5.56 ± 0.11
		(*R*_e_^−1^, *g*^iso^_1_, *g*^iso^_2_, *p*_1_, *p*_2_)	61.5 ± 2.9	142.8 ± 7.0	3.49 ± 0.17
		( 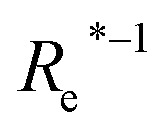 , *g*^iso^_1_, *g*^iso^_2_, *p*_1_, *p*_2_)	96.9 ± 2.9	176.0 ± 13.1	4.30 ± 0.32
		(*R*_e_^−1^, *g*^iso^_1_, *g*^iso^_2_, *p*_1_, *p*_2_, *p̄*)	67.5 ± 1.0	151.8 ± 9.5	3.71 ± 0.2
		( 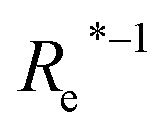 , *g*^iso^_1_, *g*^iso^_2_, *p*_1_, *p*_2_, *p̄*)	101.8 ± 5.4	188.7 ± 25.4	4.61 ± 0.62
		(*R*_e_^−1^, *g*^iso^_1_, *g*^iso^_2_, *p*_1_, *p*_2_, *ḡ*)	46.7 ± 0.6	73.4 ± 0.2	1.80 ± 0.005
		( 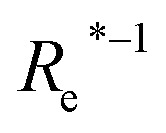 , *g*^iso^_1_, *g*^iso^_2_, *p*_1_, *p*_2_, *ḡ*)	81.0 ± 0.82	121.8 ± 0.8	2.98 ± 0.02
	LR	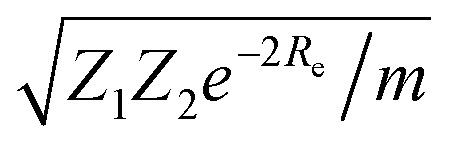	376.5 ± 6.6	529.4 ± 1.2	12.95 ± 0.03
		*R*_e_^−2^	209.6 ± 5.4	297.3 ± 1.4	7.27 ± 0.03
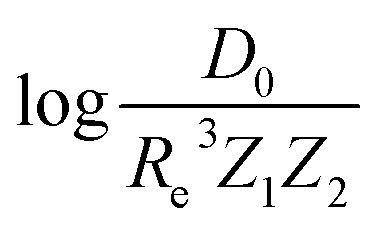	GPR	(*R*_e_, *ḡ*, *p̄*)	0.249 ± 0.008	0.357 ± 0.007	3.52 ± 0.07
		( 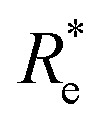 , *ḡ*, *p̄*)	0.270 ± 0.006	0.451 ± 0.007	4.45 ± 0.07
	LR	*R*_e_	0.833 ± 0.004	1.018 ± 0.014	10.03 ± 0.14

a
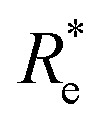
 is the predicted value from (*g*_1_, *g*_2_, *p*_1_, *p*_2_).

**Fig. 6:**
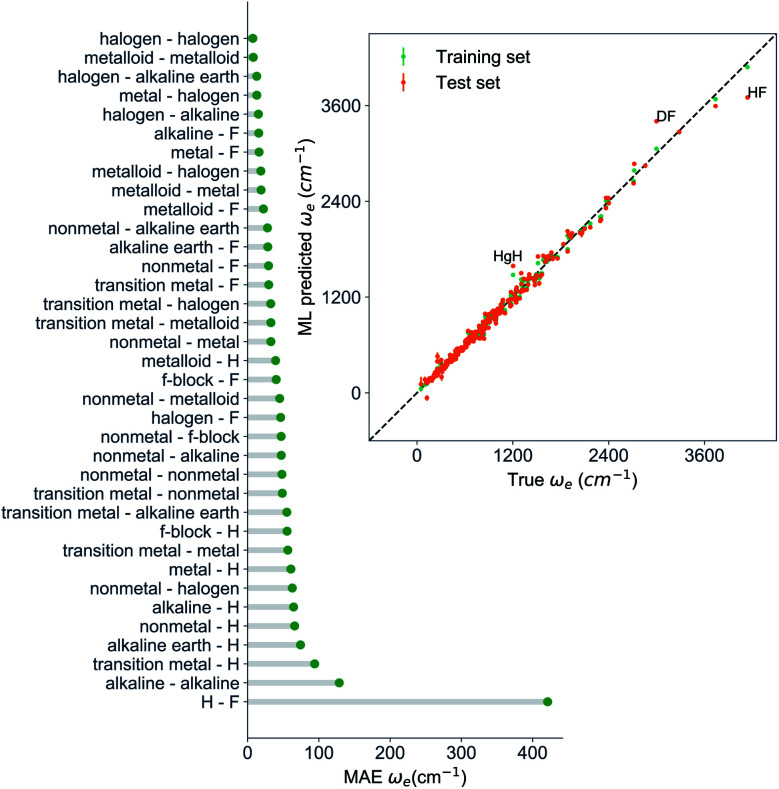
GP regression performance based on the MAE predicting *ω*_e_ for molecules in the test set using (*R*_e_^−1^, *g*^iso^_1_, *g*^iso^_2_, *p*_1_, *p*_2_, *ḡ*) as input features classified by the types of the constituent atoms. The inset shows the test set predictions of *ω*_e_ compared with respect to the true values. The values shown are the average of predictions from 1000 MC sampled training/test splittings. The GP regression model as learned from the training set gives predictions of the test and training set. Shown are the mean and standard derivation of each molecule's predictions when used as training data (green symbols) and test data (orange symbols).

In learning *ω*_e_, we find (
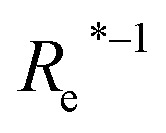
, *g*^iso^_1_, *g*^iso^_2_, *p*_1_, *p*_2_, *ḡ*) to be the best combination of features, where 
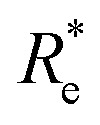
 is the predicted equilibrium distance from (*g*_1_, *g*_2_, *p*_1_, *p*_2_), *g*^iso^_*k*_ encodes the information about the hydrogen isotopes of the *k*-th atom in the molecule, and *ḡ* is the average of the groups of the two atoms. However, a much better performance is found when the true *R*_e_ value is employed. The GP model's performance is shown in the inset of Fig. 6, where it is noticed that the predicted values agree very well with the true values. Indeed, the test set MAE and RMSE are 46.7 ± 0.6 cm^−1^ and 73.4 ± 0.2 cm^−1^, respectively, while *r*_E_ = 1.80 ± 0.005%, as shown in Table 1. Despite the outstanding performance of our GPR model some molecules are still not well described as shown in Fig. 6. These outliers include HF, DF, and HgH. The large errors predicting *ω*_e_ of HF and DF can be attributed to their unique bond mechanism compared to other halogen hydrides.

Within the features (*R*_e_^−1^, *g*^iso^_1_, *g*^iso^_2_, *p*_1_, *p*_2_, *ḡ*), it is interesting that the average of groups 
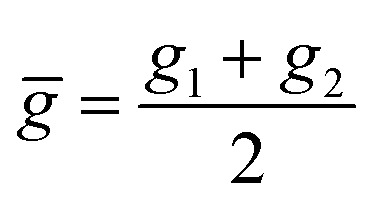
 helps in learning *ω*_e_. In particular, with *ḡ*, the MAE of the model reduces around 20% compared with the predictions using (*R*_e_^−1^, *g*^iso^_1_, *g*^iso^_2_, *p*_1_, *p*_2_) as the input feature, as summarized in Table 1. Analogously, the standard deviation of the MC training/test splittings predictions becomes much smaller, suggesting that the model is more robust for different kinds of molecules within the dataset. Actually, by introducing *ḡ*, the most significant improvement happens in the descriptions of bi-alkali molecules, where the MAE can be reduced by a factor of 3. The errors predicting HF and DF can also be reduced by a factor of 2, although they are still tricky cases for the model. On the contrary, introducing the average of periods 
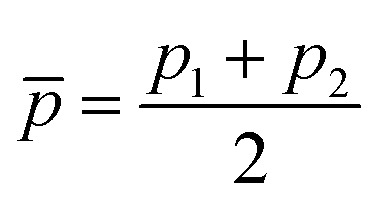
 does not help improve the model, suggesting that *ω*_e_ has a dependency on the total number of valence electrons of the two atoms rather than the number of electron shells.

**Fig. 7:**
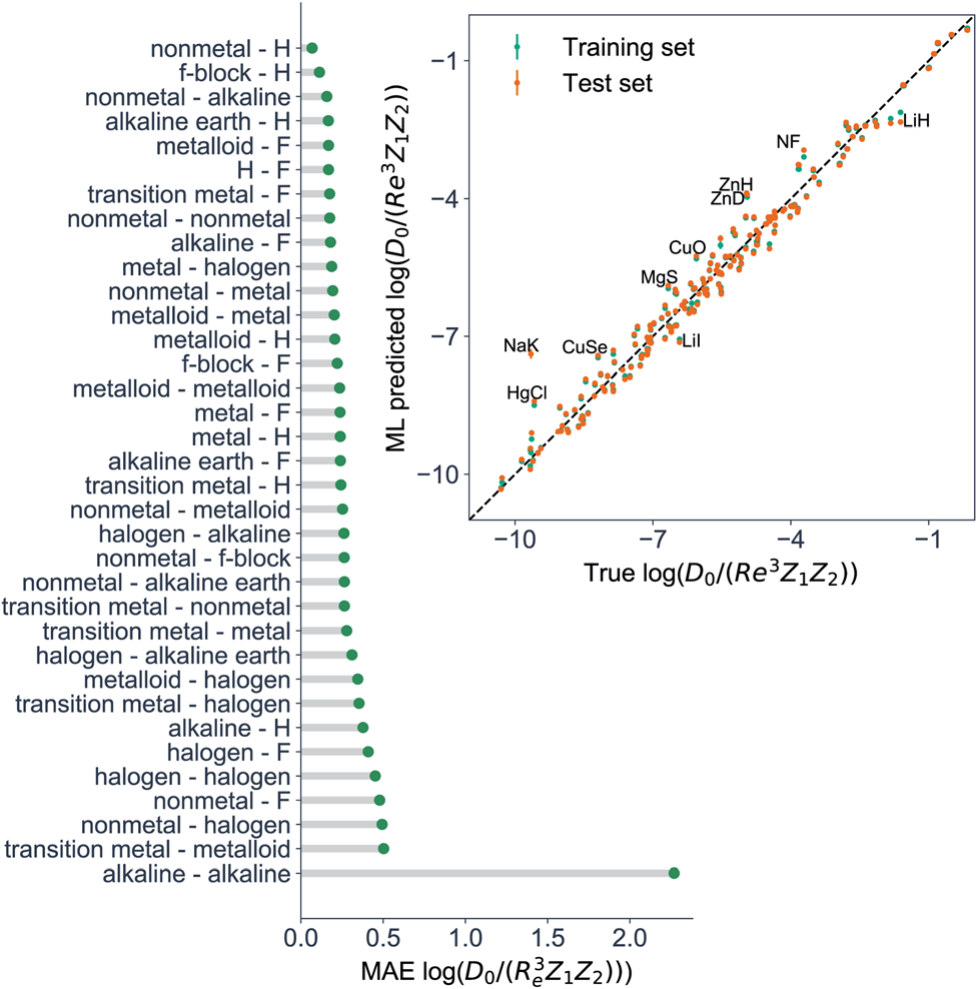
GP regression performance on predicting 
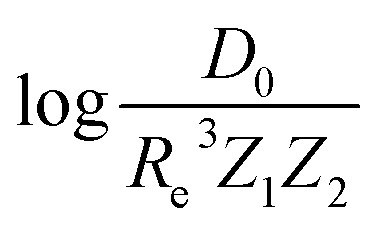
 using (*R*_e_, *ḡ*, *p̄*) as input features classified by the types of the constituent atoms. In particular, the MAE of the test set is reported. The inset shows the test set predictions of 
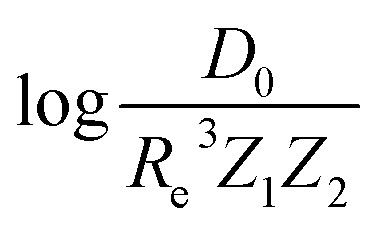
 compared with respect to the true values. The values shown are the average of predictions from 1000 MC sampled training/test splittings. The GP regression model gives predictions of the test and training set. Shown are the mean and standard derivation of each molecule's predictions when used as training data (green symbols) and test data (orange symbols).

**Fig. 8:**
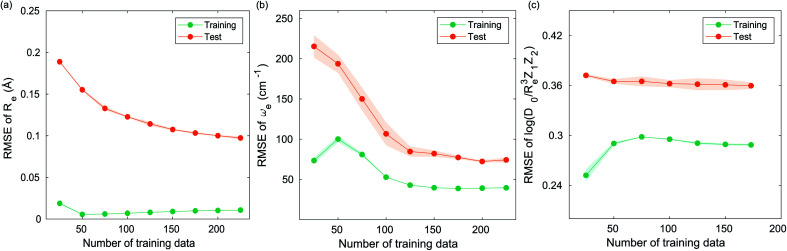
Performance of the GP regression models as a function of the training set size *N*. (a) Learning curve of *R*_e_ as a function of the size of training set, predicted with the groups and periods of the two atoms, (*g*_1_, *g*_2_, *p*_1_, *p*_2_). (b) Learning curve of *ω*_e_ as a function of the size of training set, using the equilibrium internuclear distance *R*_e_, as well as the groups and periods and the average of groups of the two atoms (*R*_e_^−1^, *g*^iso^_1_, *g*^iso^_2_, *p*_1_, *p*_2_, *ḡ*) as the input feature. (c) Learning curve of 
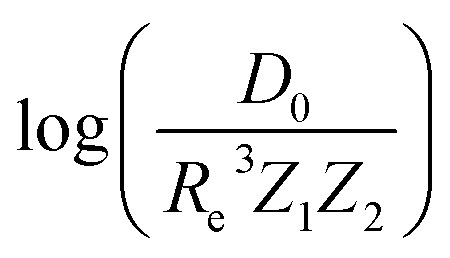
 as a function of the size of training set, using the equilibrium internuclear distance *R*_e_, as well as the averages of groups and periods of the two atoms (*R*_e_, *ḡ*, *p̄*) as the input feature. The shade around the points denotes the variance of the errors regarding the MC method.

Motivated by the pioneering work of Anderson, Parr, and coworkers,^27,29,30,45^ we study the prediction of 
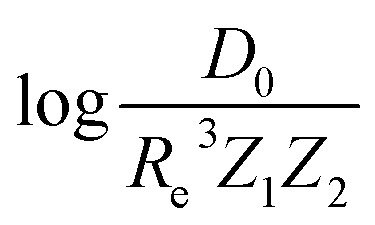
 based on GP regression and the results are shown in Fig. 7. In particular, in the figure's inset, we show the GP regression model prediction of 
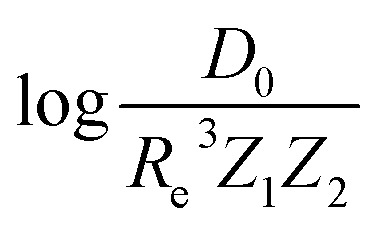
*versus* its true value, which shows a good performance with an RMSE = 0.357 ± 0.007 and a *r*_E_ equal to 3.52 ± 0.07%, as shown in Table 1. In this case, the GP is fed with (*R*_e_, *ḡ*, *p̄*) as input features and it shows a fast convergence with respect to the size of training set around *N* = 150 as shown in panel (c) of Fig. 8. The most significant outlier for 
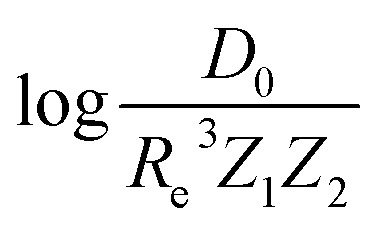
 is NaK, which is a van der Waals molecule. *D*_0_ of NaK is overestimated and it may be attributed to the fact that NaK is the only bi-alkali molecule in the dataset having *D*_0_. There are also some outliers having first-row elements and 3d transition metals.

A summary of our GP regression model performance for the different combinations of the ground state spectroscopic constants considered in this work is shown in Table 1, compared against the proposed models of Parr, Anderson *et al.*^27,29,30,45^ As a result, the GP regression model shows a superior performance against the linear model (LR in the table) based on a particular functional form of the electron density within the molecule. Indeed, the GP performance is, in some cases, five times better than the linear model (in terms of the relative error). Therefore, the group and period (correlated to the number of valence electrons and the number of electrons shells, respectively) of constituent atoms within a molecule encapsulates more valuable information regarding spectroscopic constants than using simple, functional forms for the electron density within the framework of ref. 27, 29, 30 and 45. Indeed, it is interesting to notice that, when predicting *R*_e_ and *ω*_e_, one needs groups and periods of each atom in the molecule, whereas 
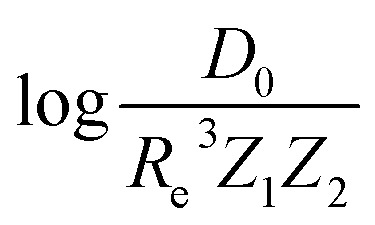
 can be well described only with the average of group and period of the two atoms. Therefore, 
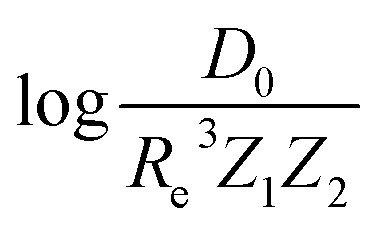
 is correlated to groups and periods' additive properties rather than the differences between the two atoms caused by their different groups.

To further examine if our ML approach is generalizable, we have selected 26 molecules out of the dataset and unseen by the ML algorithm including CoO,^56^ CrC,^57^ InBr,^58^ IrSi,^59^ MgD,^60^ MoC,^61^ NbC,^61^ NiBr,^62^ NiC,^63^ NiO,^64^ NiS,^65^ PbI,^66^ PdC,^61^ RuC,^61^ RuF,^67^ ScBr,^62^ SnI,^66^ TiBr,^62^ UF,^68^ UO,^69^ WC,^70^ YC,^61^ ZnBr,^62^ ZrC,^61^ ZrCl,^71^ ZrF.^71^ The MAE of the GP regression model predicting ground state *R*_e_ of the extra test set is 0.066 Å. The average relative error (defined as the absolute errors of each molecule divided by their true *R*_e_) is 3.3%. Indeed, for CrC, InBr, MgD, ZnBr, ZrCl the relative errors are <1%. Within this extra test set, experimental ground state *ω*_e_ values are also available for 14 molecules: InBr, MoC, NbC, NiC, NiO, NiS, PbI, PdC, RuC, SnI, UO, WC, YC and ZnBr. The MAE of GPR model predictions is 30 cm^−1^ (4%). For RuC and ZnBr, the relative errors are below 1%, and for NiS and MoC, the relative errors are below 2%. For MoC, NbC, PbI, SnI, YC and ZrC, the experimental binding energy has been reported and the MAE of our GPR model to predict *D*_0_ is 0.32 eV (7.6%). Therefore, our models perform fairly well in this extra test set.

### 5.2 Learning the first excited state spectroscopic constants

**Fig. 9:**
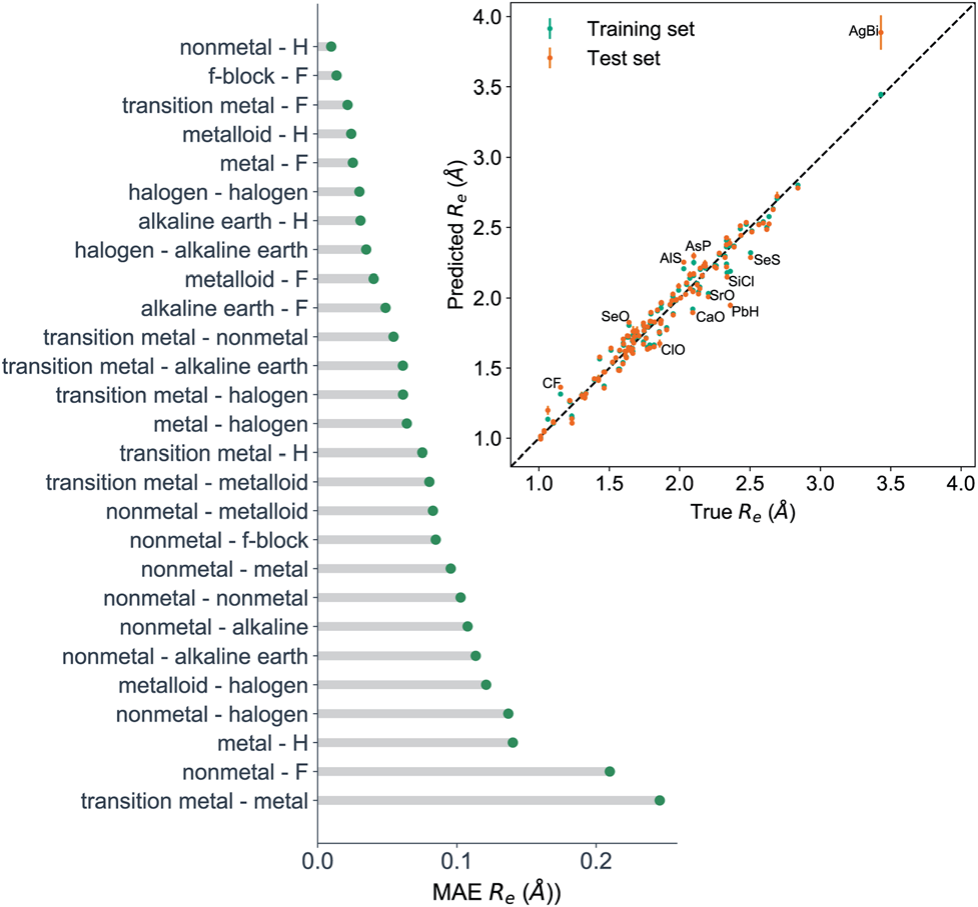
The test set MAE predicting A excited electronic state *R*_e_ by GP regression, using (*g*_1_, *g*_2_, *p*_1_, *p*_2_, *R*_e_(X), *D*(IP, EA)) as input features, classified by the types of the constituent atoms. The inset shows the test set predictions of the A-excited electronic state *R*_e_ compared with respect to the true values. The values shown are the average of predictions from 1000 MC sampled training/test splittings. The GP regression model as learned from the training set gives predictions of the test and training set. Shown are the mean and standard derivation of each molecule's predictions when used as training data (green symbols) and test data (orange symbols).

To learn the equilibrium internuclear distance *R*_e_ of the A excited electronic state for different molecules, we need to employ atomic features of the two constituent atoms, including *g*_1_, *g*_2_, *p*_1_, *p*_2_, *D*(IP, EA), and the ground state *R*_e_(X) when constructing the GP regression models. It is interesting that including *D*(IP, EA) can improve the predictions (Table 2), which is defined as
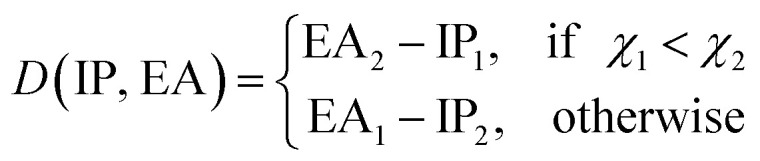
where IP_*i*_, EA_*i*_ and *χ*_*i*_ are the ionic potential, electron affinity and electronegativity of atom *i*, respectively. Therefore, *D*(EA, IP) qualitatively measures the electron transfer between the two constituent atoms. The resultant test set MAE, RMSE and *r*_E_ are 0.0691 ± 0.0062\AA, 0.098 ± 0.0097\AA, 5.32 ± 0.53, respectively. As shown in Fig. 9, similar to the results of ground state *R*_e_, the transition metal–metal compounds are the most difficult ones to predict.

**Table 2:** Regression model predictions of the A excited electronic state *R*_e_ and *ω*_e_. *g*_*i*_ and *p*_*i*_ are the groups and periods of the *i*-th atom, respectively whereas *g*^iso^_*i*_ stand for the group encoding the information of isotopes of hydrogen. *p̄*, *ḡ* are the average of groups and periods of the two atoms, respectively. *R*_e_(X) and *R*_e_(A) refer to the ground state and A-state *R*_e_, respectively. *ω*_e_(X) refers to the ground state *ω*_e_

Property	Model	Feature	Test MAE	Test RMSE	Test *r*_E_ (%)
*R*_e_ (Å)	GPR	(*R*_e_(X), *g*_1_, *g*_2_, *p*_1_, *p*_2_)	0.0783 ± 0.0018	0.107 ± 0.0026	5.81 ± 0.14
		(*R*_e_(X), *g*_1_, *g*_2_, *p*_1_, *p*_2_, *D*(IP, EA))	0.0691 ± 0.0062	0.098 ± 0.0097	5.32 ± 0.53
*ω*_e_ (cm^−1^)	GPR	(*ω*_e_(X), *R*_e_^−1^(X), *R*_e_^−1^(A), *g*^iso^_1_, *g*^iso^_2_, *p*_1_, *p*_2_, *ḡ*)	71.8 ± 1.4	107.9 ± 4.4	11.3 ± 0.46
		(*ω*_e_(X), *R*_e_^−1^(X), *R*_e_^−1^(A), *g*^iso^_1_, *g*^iso^_2_, *p*_1_, *p*_2_)	70.4 ± 0.9	105.1 ± 1.5	11.0 ± 0.15
		(*ω*_e_(X), *R*_e_^−1^(X), *R*_e_^−1^(A), *g*_1_, *g*_2_, *p*_1_, *p*_2_)	70.6 ± 0.9	105.1 ± 1.1	11.0 ± 0.12

**Fig. 10:**
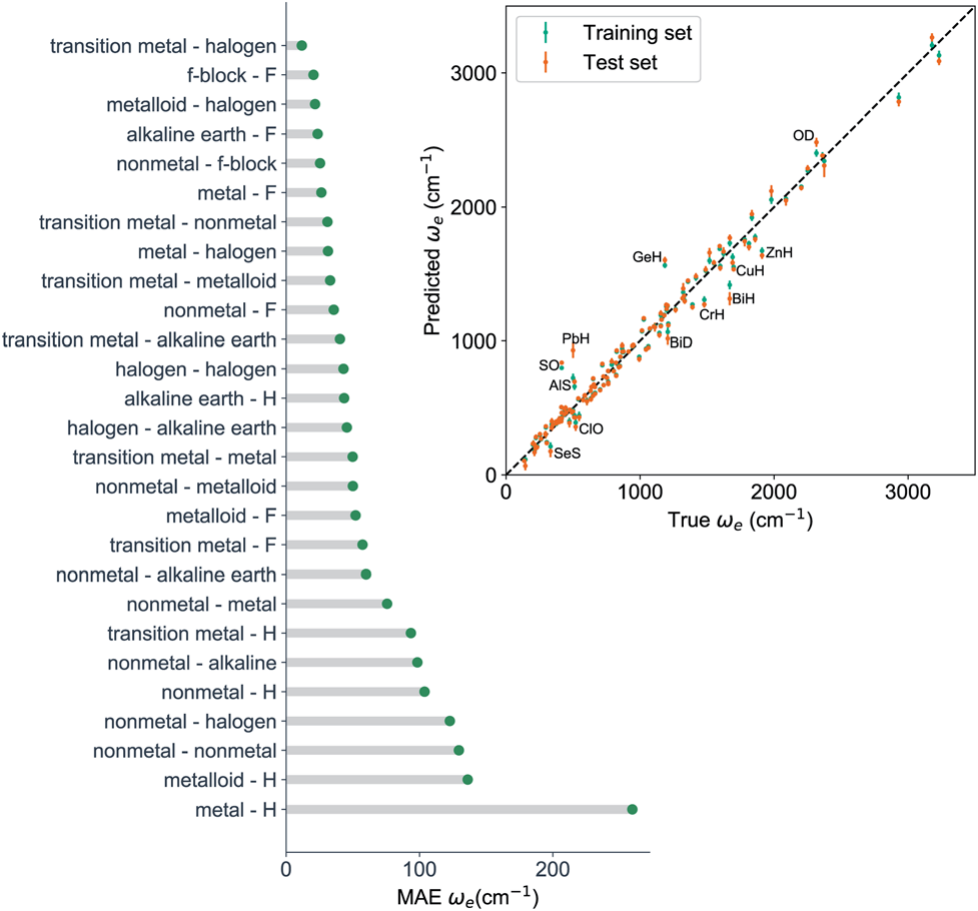
The test set MAE predicting A excited electronic state *ω*_e_ by GP regression, using (*ω*_e_(X), *R*_e_^−1^(X), *R*_e_^−1^(A), *g*_1_, *g*_2_, *p*_1_, *p*_2_) as input features, classified by the types of the constituent atoms. The inset shows the test set predictions of A-excited electronic state *ω*_e_ compared with respect to the true values. The values shown are the average of predictions from 1000 MC sampled training/test splittings. The GP regression model as learned from the training set gives predictions of the test and training set. Shown are the mean and standard derivation of each molecule's predictions when used as training data (green symbols) and test data (orange symbols).

For learning *ω*_e_ of the A excited electronic state, in addition to the ground state *R*_e_^−1^(X), it is also necessary to include the A state *R*_e_^−1^(A). Furthermore, it is better to include the ground state *ω*_e_(X) as the input feature. The results are shown in Fig. 10 in which (*ω*_e_(X), *R*_e_^−1^(X), *R*_e_^−1^(A), *g*_1_, *g*_2_, *p*_1_, *p*_2_) leads to a RMSE of 105.1 ± 1.1 cm^−1^ and *r*_E_ = 11.0 ± 0.12%. We also find that including the average of groups *ḡ* or the isotope information cannot further improve the model performance. This is expected, since this information have already been encoded in the ground state *ω*_e_.

The performance of our models predicting the A excited electronic state *R*_e_ and *ω*_e_ are summarized in Table 2. Compared to the ground state predictions, the errors predicting the A excited electronic state spectroscopic constants are around two times larger, suggesting the difficulty predicting the excited state properties. However, we notice that *ω*_e_ is correlated with the inverse of *R*_e_(A) as for ground state molecules. Our findings corroborate the hypothetical relationship between *R*_e_ and *ω*_e_ in the early times of molecular spectroscopy as it has been introduced in Section 2.

## 6 Conclusions

In summary, we have shown that using the GP regression model, the main spectroscopic constants of diatomic molecules are related. This result confirms the scenario that Kratzer and Mecke envisioned a century ago.^2,3^ The relationships are mostly independent of the nature of the chemical bond of the diatomic molecule. In particular, we have demonstrated that merely using the atoms' group and the period within a molecule as input features can predict particular combinations of spectroscopic constants with an error *r*_E_ < 5%. In other words, the spectroscopic constants of diatomic molecules can be efficiently learned from an appropriate dataset by GP regression models, and their values can be accurately predicted. Furthermore, we have shown that GP regression can efficiently learn spectroscopic relationships for excited electronic states of molecules with an error *r*_E_ < 11%.

Despite the present GP models' outstanding performance, machine learning methods may be considered mere fitting techniques or as a black-box algorithm that one can hardly learn anything new from them. This statement is not accurate. As an example, here, we emphasize what we have learned from the present machine learning approach:

• It is generally assumed that some molecular properties can be predicted based on the forming atom's positions in the periodic table.^72^ However, the predictions are only qualitative rather than quantitative. For instance, it is possible to anticipate the nature of a molecule's bond, but it cannot accurately guess its dissociation energy. However, thanks to ML, we know that it is possible to predict reasonably accurate spectroscopic constants using the constituent atoms' group and period.

• We have learned that *ω*_e_ and *R*_e_ depend strongly on the number of valence electrons and electrons shells of the atoms forming a molecule, whereas the average number of valence electrons also plays an important role in describing *ω*_e_. 
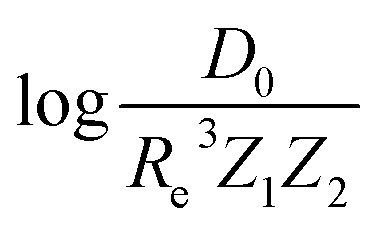
 depends on the average number of valence electrons and average number of electron shells of the molecule.

• The capability of learning excited electronic state properties of diatomic molecules may open the possibility of predicting Franck–Condon factors for interesting transitions regarding direct cooling of molecules.^47,73–76^

Finally, we would like to emphasize that there are around 7000 heteronuclear molecules, and we only utilize 256 of these for our GP regression model. The limited availability of spectroscopic data (only around 3% of possible heteronuclear diatomic molecules) shows the vast amount of spectroscopy that can be done within the realm of diatomic molecules. The more data we have, the more accurate will be the GP regression model predictions before reaching convergence of the learning curve, and the more knowledgeable the community will be about the fundamental properties of diatomic molecules. From our perspective, the present work may motivate data science-driven studies on the field of spectroscopy of diatomic molecules. In particular, it will help to evolve the field of spectroscopy towards the current information era and help to achieve a better understanding on the spectroscopic properties. Furthermore, our results may also bring some insight for the development of features and geometry representations in material science.

## Appendix: details about the GP regression models

The choice of covariance functions defines the smoothness of the data points. In learning *R*_e_, the covariance function employed is the exponential kernel defined as14
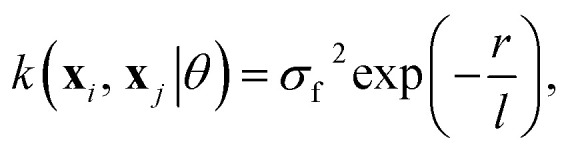
where *σ*_f_ is the signal variance, *l* is the characteristic length scale, and *r* is the Euclidean distance between **x**_*i*_ and **x**_*j*_.

In learning *ω*_e_, we use the Matérn class of covariance functions^40^15
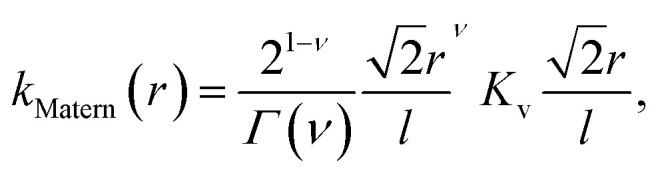
with *ν* = 5/2. *K*_v_ is modified Bessel function in *D* dimensions, *r* is the Euclidean distance between *x* and *x*′, then the Matern 5/2 kernel function is16
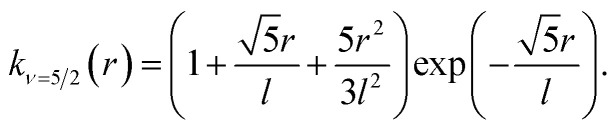


The explicit basis functions in learning *R*_e_ are linear basis, while when learning *ω*_e_ and 
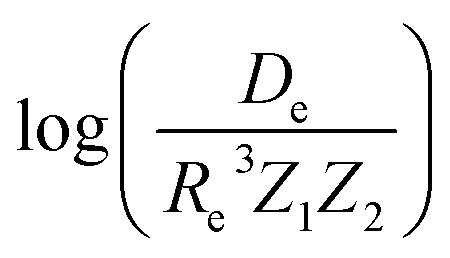
 the basis functions are set to be constant.

## Conflicts of interest

There are no conflicts to declare.

## Supplementary Material
